# Automated landmark-based mid-sagittal plane: reliability for 3-dimensional mandibular asymmetry assessment on head CT scans

**DOI:** 10.1007/s00784-025-06397-z

**Published:** 2025-05-26

**Authors:** Sophie Alt, Laurent Gajny, Françoise Tilotta, Thomas Schouman, Gauthier Dot

**Affiliations:** 1 Institut de Biomecanique Humaine Georges Charpak, Arts et Metiers Institute of Technology, Paris, France; 2 Université Paris Cité and Sorbonne Paris Nord, UMR1333 INSERM Santé Orale, Montrouge, F-92120 France; 3 Service de Chirurgie maxillo-faciale, Sorbonne Université, AP-HP, Hôpital Pitié Salpêtrière, 83 boulevard de l’Hôpital, Paris, 75013 France; 4 Service Médecine Bucco-Dentaire, AP-HP, Hôpital Pitié-Salpêtrière, Paris, F-75013 France

**Keywords:** Orthodontics, Orthognathic surgery, Surgery, Computer-assisted, Anatomic landmarks, Facial asymmetry, Deep learning

## Abstract

**Objective:**

The determination of the mid-sagittal plane (MSP) on three-dimensional (3D) head imaging is key to the assessment of facial asymmetry. The aim of this study was to evaluate the reliability of an automated landmark-based MSP to quantify mandibular asymmetry on head computed tomography (CT) scans.

**Materials and methods:**

A dataset of 368 CT scans, including orthognathic surgery patients, was automatically annotated with 3D cephalometric landmarks via a previously published deep learning-based method. Five of these landmarks were used to automatically construct an MSP orthogonal to the Frankfurt horizontal plane. The reliability of automatic MSP construction was compared with the reliability of manual MSP construction based on 6 manual localizations by 3 experienced operators on 19 randomly selected CT scans. The mandibular asymmetry of the 368 CT scans with respect to the MSP was calculated and compared with clinical expert judgment.

**Results:**

The construction of the MSP was found to be highly reliable, both manually and automatically. The manual reproducibility 95% limit of agreement was less than 1 mm for -y translation and less than 1.1° for -x and -z rotation, and the automatic measurement lied within the confidence interval of the manual method. The automatic MSP construction was shown to be clinically relevant, with the mandibular asymmetry measures being consistent with the expertly assessed levels of asymmetry.

**Conclusion:**

The proposed automatic landmark-based MSP construction was found to be as reliable as manual construction and clinically relevant in assessing the mandibular asymmetry of 368 head CT scans.

**Clinical relevance:**

Once implemented in a clinical software, fully automated landmark-based MSP construction could be clinically used to assess mandibular asymmetry on head CT scans.

## Introduction

The use of three-dimensional (3D) computed tomography (CT) or cone beam computed tomography (CBCT) scans in patients with complex maxillomandibular deformities and craniofacial anomalies has increased greatly because it has been shown to improve diagnosis and treatment planning [1, 2]. In particular, 3D scans are now routinely used for planning computer-assisted orthognathic surgery. Several methods have been proposed to perform virtual planning, but most of them are based on the same initial steps: (A) segmentation of the facial units, such as cranial base, maxilla, mandible, and teeth; (B) annotation with cephalometric landmarks; (C) orientation of the scans; and (D) quantitative evaluation of the asymmetry of the facial units [3].

The determination of the mid-sagittal plane (MSP) is the key element of the scan orientation, as this plane will guide all asymmetry measurements of the facial units. To date, three methods based on cephalometric landmarks, morphometry, and symmetry plane have been proposed to construct the MSP. A recent systematic review showed that no method was more efficient than the other methods were [4]. When the cephalometric approach is used, this review recommends to define the MSP as a plane passing through the median nasion and sella landmarks and perpendicular to the Frankfurt horizontal plane (FHP), as suggested in previous clinical evaluation studies [5, 6]. The main disadvantage of the cephalometric approach over the other two methods is the need for landmark localization, which classically relies on operator-dependent, time-consuming manual localization.

To relieve clinicians from this tedious task, automated deep learning methods have shown promising results, raising hopes for full automation of the landmarking process [7]. However, the same error patterns have been found between manually and automatically localized landmarks [8]. To our knowledge, the use of 3D automated landmarking for MSP construction has not been investigated.

One way to assess the position of each facial unit is to construct a local coordinate system (LCS) for each unit and compare its orientation to the world coordinate system (WCS) of the skull base, which is aligned with the MSP [9]. For an ideally symmetrical patient, the entire coordinate system is aligned along the patient’s MSP. For a patient with some asymmetry, measuring the translations and rotations between the LCS and WCS provide a quantitative assessment of the asymmetry of each facial unit.

This paper focuses on the assessment of mandibular asymmetry with respect to the MSP, which remains a clinical challenge with no widely accepted reference method [4]. The main objective of this study was to evaluate the reliability of automated 3D landmark-based MSP construction to quantify mandibular asymmetry on head CT scans.

## Materials and methods

### Dataset

Our dataset consisted of 368 anonymized whole-head CT scans based on two retrospective samples described in previous studies [10–12]. These two samples consisted of: (A) 79 volunteers without any marked dento-maxillo-facial deformities and (B) 289 preorthognathic surgery patients with dento-maxillo-facial deformities. The characteristics of the dataset are detailed in Table 1. This study was approved by an appropriate institutional review board (IRB No. CRM-2001-051).

**Table 1:** Dataset characteristics

	Volunteers subjects	Presurgical subjects
Number of CT scans	79	289
Age, mean ± SD, years	31 ± 13	26 ± 8
Gender, no. (%)		
Female	53 (67)	162 (56)
Male	26 (33)	127 (44)
Mean in-plane pixel size (mm^2^)	0.48 * 0.48	0.45 * 0.45
Mean slice thickness (mm)	0.7	0.34
Number of scans by CT Machine		
GEHC Discovery CT750 HD		271
GEHC Optima CT540 or CT660		9
Siemens SOMATOM Sensation 16	79	
Other CT Machine^1^		9

GEHC: GE Healthcare. ^1^GEHC Revolution CT, Philips Ingenuity Core, Siemens SOMATOM Definition AS, Toshiba Aquilion Prime SP

### Deep learning–based landmarking

Our dataset was automatically annotated following a previously published deep learning-based approach [8]. This approach relies on a Spatial Configuration-Net (SCN) model trained in a coarse-to-fine manner on 160 head CT scans for the localization of 33 cephalometric landmarks. Those 160 training CT scans were independent from our dataset. Evaluation of the model on a hold-out test set of 37 CT scans showed that its reliability for skeletal assessment was comparable to that of clinicians [8]. The reported mean localization error was 1.0 ± 1.3 mm, while the success detection rates for 2.0, 2.5, and 3.0 mm were 90.4%, 93.6%, and 95.4%, respectively [8]. Inference (prediction made by the trained model) was performed following the instructions of the authors, and landmark coordinates were automatically exported in a csv file. Inference required around 1 min per CT scan on our laboratory workstation (CPU AMD Ryzen 9 3900 × 12-Core; 128 Gb RAM; GPU Nvidia Titan RTX 24Gb).

### MSP construction

Five landmarks (Table 2) were used to define a WCS aligned with the FHP and MSP.

**Fig. 1:**
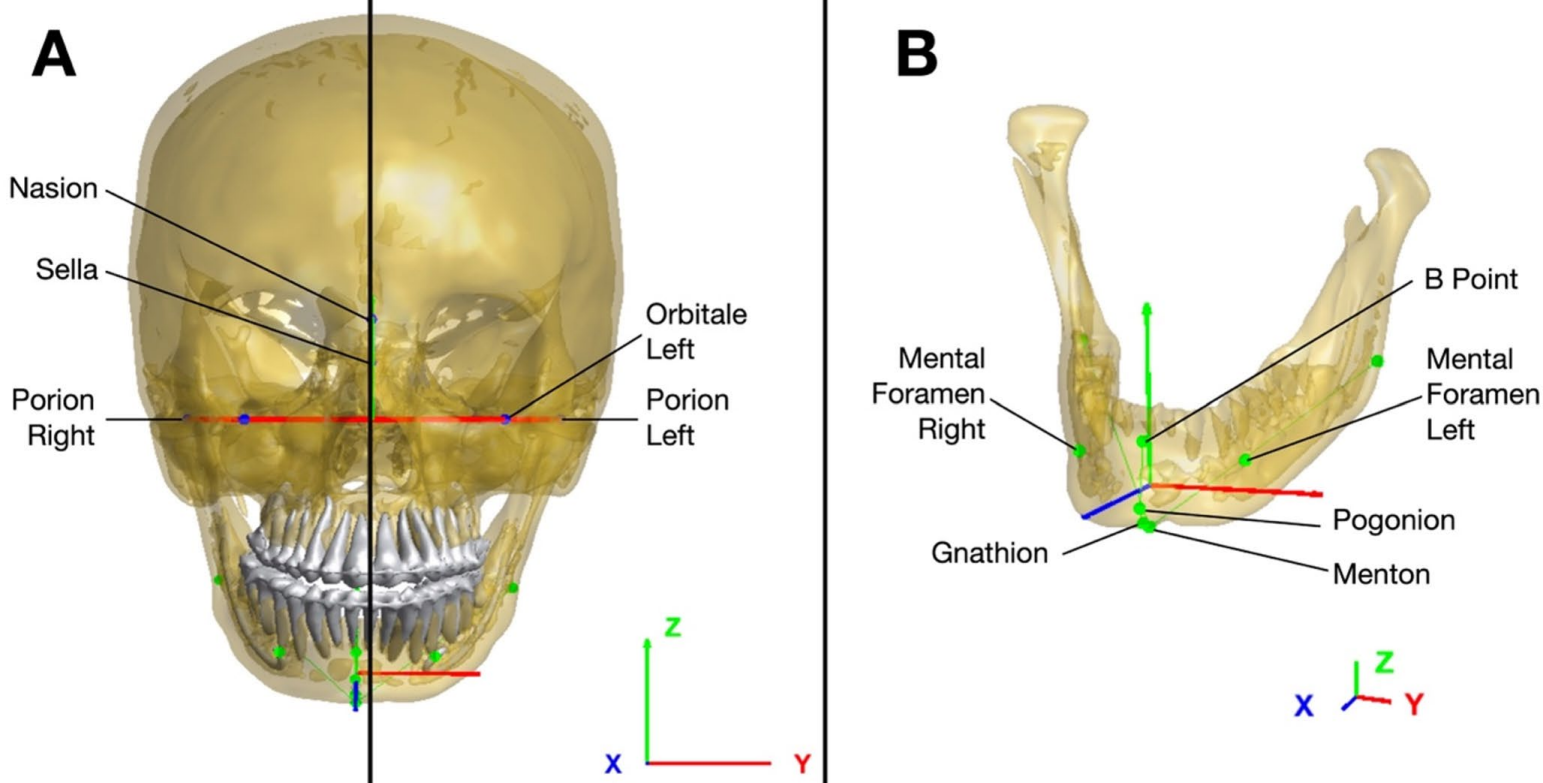
3D model of a CT scan after coordinate system construction, and landmarks names. **A**: WCS and LCS in frontal view; **B**: Mandible LCS

Three of these landmarks (porion left, porion right, orbitale left) were used to construct the FHP. The z-axis of the WCS was defined as normal to the FHP. The x-axis was calculated as the vector from the projection of the Sella (P1) to the projection of the Nasion (P2) on the FHP. The y-axis was then calculated as the cross product of the x-axis and the z-axis. The origin of the WCS was the projection of the nasion on the FHP (P1). All CT scans in the database were aligned to the WCS (Fig. 1A).

**Table 2:** Definition of landmarks used for WCS construction

Landmark name	Description
Nasion (Na)	Medial (and upper) point of the frontonasal suture
Orbitale Left (Or-L)	Lowest point of the left orbital rim
Porion Left (Po-L)	External & uppermost point of the left auditory canal
Porion Right (Po-R)	External & uppermost point of the right auditory canal
Sella (S)	Central point of the sella

### Mandibular asymmetry measurement

The mandibular LCS was defined using 6 landmarks (Table 3). The origin of this coordinate system was the barycenter of these 6 points. The z-axis was defined by the normed vector between the barycenter of the pogonion, gnathion, and menton landmarks and the B point. A temporary y-axis was constructed owing to the normalized vector between the right and left foramen. The x-axis was calculated via the cross product of the latter two vectors. Finally, the orientation of the y-axis was refined by taking the cross product of the x- and z-axes, ensuring orthogonality and proper alignment within the coordinate system (Fig. 1).

The mediolateral translation (y-axis, frontal asymmetry) of the mandible, as well as the rotations (x-axis– roll, and z-axis– yaw), were computed and reported as measurements of mandibular asymmetry.

**Table 3:** Definition of landmarks used for mandible LCS construction

Landmark name	Description
B Point (B)	Medial and most posterior point of the mandible
Pogonion (Pog)	Medial and most anterior point of the mandible
Gnathion (Gn)	Medial and midpoint between Pog and Me
Menton (Me)	Medial and lowest point of the mandible
Mental Foramen Left (MF-L)	External & most mesial point of the left mental foramen
Mental Foramen Right (MF-R)	External & most mesial point of the right mental foramen

### Reliability of manual and automatic MSP construction

To compare automatic and manual MSP construction, we conducted a repeatability and reproducibility (R&R) study of manual MSP construction. We conducted a sample size calculation to guarantee 15% uncertainty in the repeatability or reproducibility outcome of six repetitions [13]. Thus, this study required a sample size of at least 17 participants. Manual localization of the 5 landmarks used to construct the MSP was performed twice by 3 operators on a random selection of 19 CT scans from patients before orthognathic surgery. These localizations were performed as part of a previous study published by our research team [14].

The 19 CT scans were aligned following those manually constructed WCS, and mandible asymmetry (translation along the y-axis and rotations along the x- and z-axes) was measured for each repetition. The landmarks used for the mandible LCS construction were those automatically localized as described above. For each measurement, R&R standard deviations (SD) were computed according to the ISO 5725 standard of the International Organization for Standardization [15]. The Bland–Altman 95% limits of agreement (LoA) were computed considering a 95% confidence interval (CI) of 2 × SD of repeatability and reproducibility. Modified Bland-Altman plots, showing the deviations of the measurements from their means for the 19 CT scans, were computed for each measurement [16, 17].

The mandibular asymmetry based on the automatic MSP construction was then compared with the 95% LoA of the manual measurement.

### Clinical relevance of MSP construction

The level of mandibular asymmetry with respect to the MSP was evaluated qualitatively as none / moderate / severe for each subject in our dataset by an experienced orthodontist. This classification was based on the observation of the CT scan segmentations after manual reorientation of the data to align it with the FHP and MSP on the 3DSlicer software (version 5.6.1 - http://www.slicer.org/) [18]. The operator was blinded to the measurements, and no additional quantitative analysis was performed.

Following this method, all healthy volunteers were evaluated as symmetric, and presurgical subjects were divided in 3 groups depending on their asymmetry level. Our dataset was divided into 4 groups: (A) symmetric healthy volunteers (*n* = 79); (B) presurgical symmetric subjects (*n* = 191); (C) presurgical moderately asymmetric subjects (*n* = 77); and (D) presurgical severely asymmetric subjects (*n* = 21). The measures of mandible asymmetry were compared among the four groups via an appropriate ANOVA method followed by pairwise comparison.

All the data were analyzed via MATLAB software (v.R2022a, MathWorks, Natick, MA, USA) and RStudio (v.1.3, RStudio PBC, Boston, MA, USA).

## Results

### Reliability of manual and automatic MSP construction

**Fig. 2:**
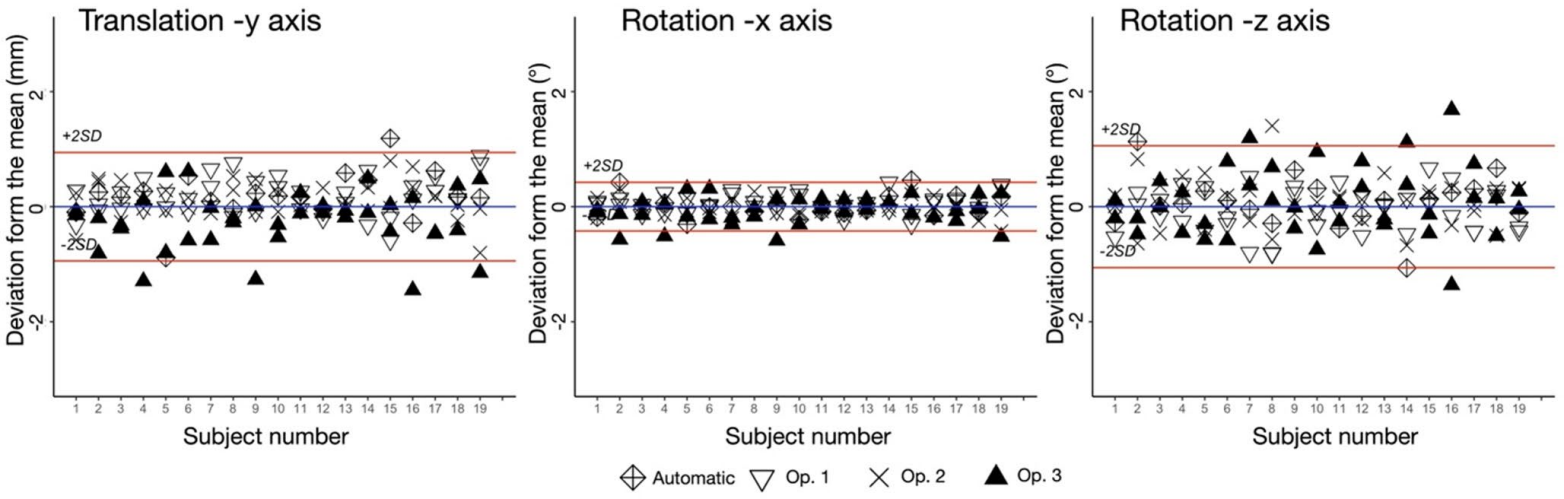
Bland-Altman plots for mandibular asymmetry measurements, showing the deviations from the mean (blue line) of the 6 repetitions for the 19 subjects and automatic measurements. The red lines represent the ± 2 × SD of reproducibility (LoA). SD, standard deviation

When the MSP was constructed manually, the 95% LoA of the mandibular asymmetry measures were inferior to 1 mm for translation along the y- axis and inferior to 1.1° for rotations along the x- and z-axes (Table 4). Figure 2 shows the modified Bland-Altman plots for those 3 measurements.

When comparing mandibular asymmetry measures between automatic and manual MSP constructions (based on the means of manual annotations), the mean differences in the automatic measurements were inferior to 0.4 mm or 0.4° (Table 4). When automatic measurements were compared with manual measurements, between 89% and 95% of them were within 95% LoA (Table 4). The results of the automatic measurements are plotted in Fig. 2.

**Table 4:** Reliability of manual and automatic MSP construction for mandibular asymmetry measurement

	Manual MSP construction		Automatic MSP construction	
	Repeatability 95% LoA	Reproducibility 95% LoA	Mean error ± SD	Within reproducibility 95% LoA
Mandible Translation -Y (mm)	0.78	0.94	0.34 ± 0.31	95% (*n* = 18)
Mandible Rotation -X (°)	0.36	0.42	0.15 ± 0.13	95% (*n* = 18)
Mandible Rotation -Z (°)	0.94	1.06	0.33 ± 0.32	89% (*n* = 17)

### Clinical relevance of MSP construction

**Fig. 3:**
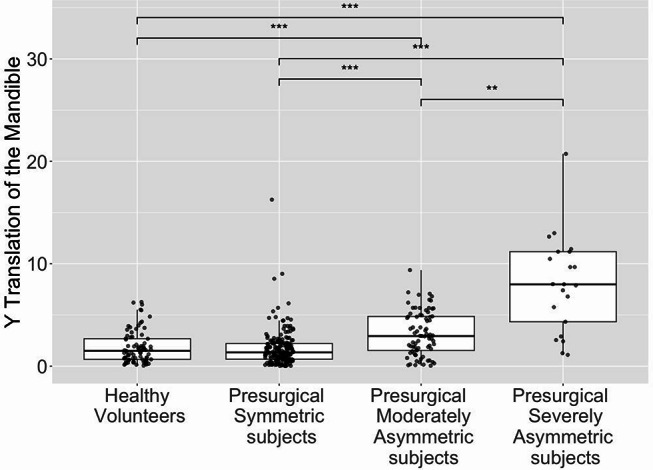
Distribution of mandible translation along–y measures according to qualitative asymmetry level and statistical test results. **p ≤ 0.01; ***p ≤ 0.001

**Fig. 4:**
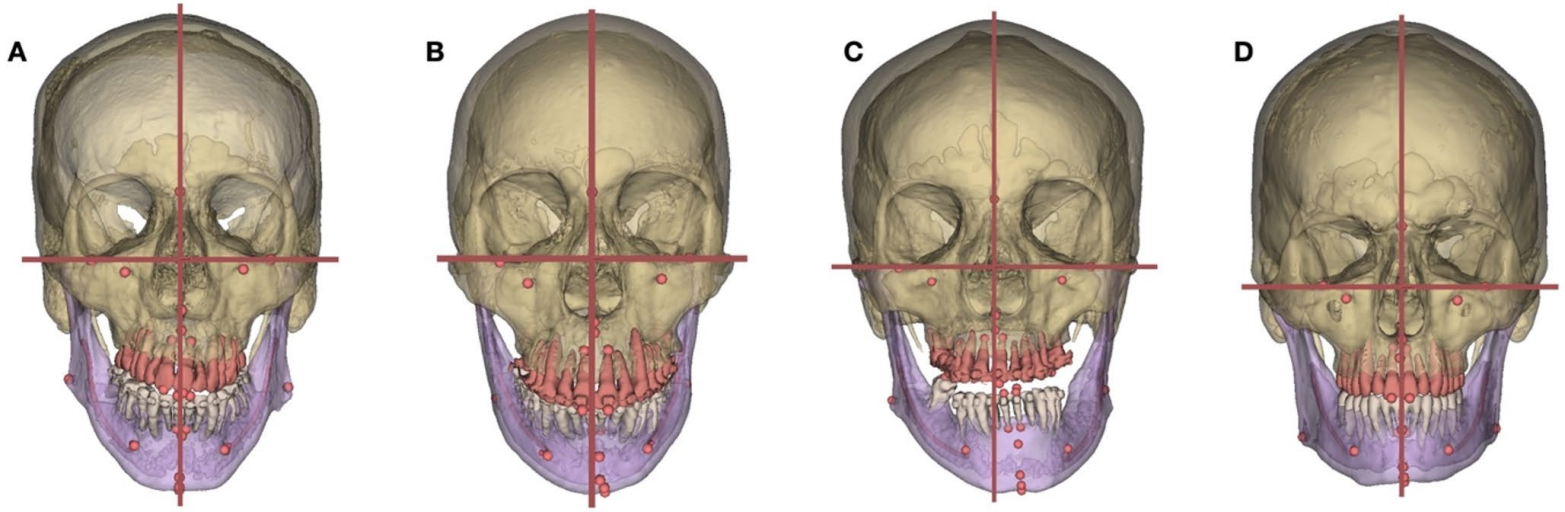
3D models of CT scans representative of our 4 groups. **A**: Presurgical symmetric subject; **B**: Presurgical moderately asymmetric subject; **C**: Presurgical severely asymmetric subject; **D**: Symmetric volunteer

Considering the non-normality of some of our samples, we performed a Kruskal-Wallis test. Significant differences between at least 2 groups (*p* < 0.01) were found for each measure: y-axis translation, x- and z- axes rotation. Figure 3 illustrates the mandible y-axis translation measures according to the asymmetry level and shows the statistical test results. All the groups were significantly different from one another in terms of y-axis translation, except for symmetric volunteers and presurgical symmetric subjects. Figure 4 illustrates our group repartition with 3D models of four representative subjects.

### Qualitative analysis of failure cases

**Fig. 5:**
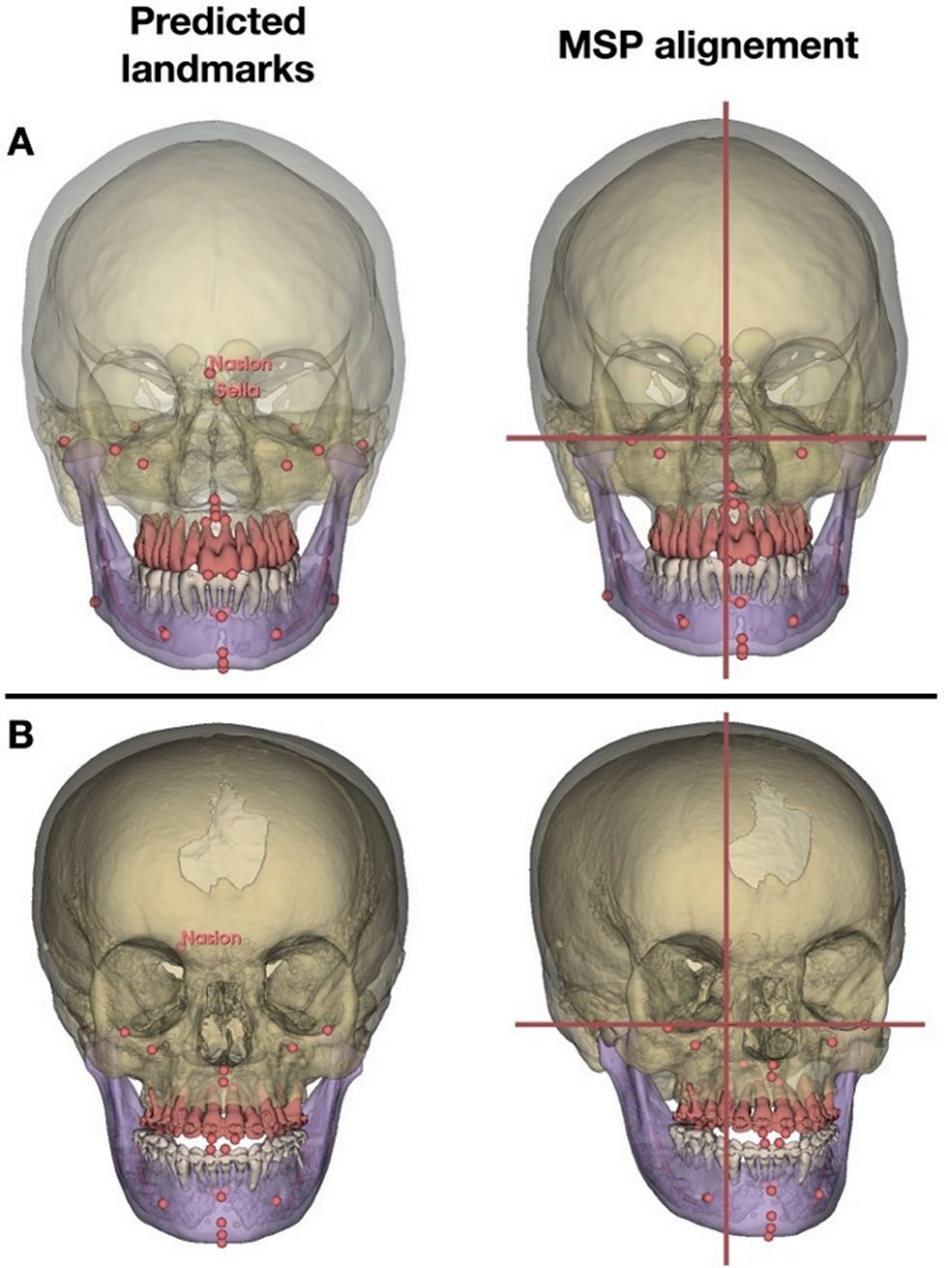
3D models of 2 failures cases, showing predicted landmarks (left side) and MSP alignment (right side). **A**: Volunteer subject with nasion and sella landmarks slightly displaced laterally. **B**: Presurgical symmetric patient with cleidocranial dysplasia, misplaced Nasion landmark

Two failure cases, selected as two symmetric subjects with reported significant mandibular translation along the -y axis, were analyzed qualitatively. Figure 5A shows a volunteer subject in whom the nasion and sella landmarks were slightly displaced laterally, resulting in a faulty MSP alignment and a -y mandibular translation of 5.5 mm. Figure 5B shows a presurgical symmetric patient with cleidocranial dysplasia where landmark nasion was misplaced, likely due to the atypical morphology of the upper skull. This error resulted in an incorrect MSP alignment and a -y mandibular translation of 16.2 mm.

## Discussion

The construction of the MSP is a recurring clinical issue in orthodontic and orthognathic planning, as this plane is key to assessing the asymmetry of facial units. In this study, we measured the reliability and clinical relevance of an automatic landmark-based MSP. This evaluation was based on measurements of mandibular asymmetry in 368 CT scans. The MSP construction was shown to be highly reliable, both manually and automatically. The manual reproducibility 95% LoA was inferior to 1 mm for y-axis translation and inferior to 1.1° for x- and z-axes rotations, and the automatic measurement showed errors within these 95% LoA. The automatic MSP construction was shown to be clinically relevant, as the measures were consistent with the expertly assessed levels of asymmetry.

Our results concerning manual reproducibility are in line with several reports in the literature, indicating that MSP construction on the basis of landmarks is highly reproducible [5, 6, 19]. To our knowledge, the reliability of automatic landmark-based MSP construction had not yet been studied. Automatic head (CB)CT landmarking is still in its early stages, but the first research reports have shown very promising accuracy results [7]. The fact that our automatic landmarking method, which is based on a coarse-to-fine SCN network, is shown to be reliable for MSP construction is an important finding to prove the clinical interest of automatic 3D cephalometry.

To evaluate the clinical relevance of our automatic MSP construction, we automatically annotated many CT scans to quantify mandibular asymmetry. Given that there is no “ground truth” of asymmetry assessment, we compared automatic measurement to an expert clinical evaluation. This approach proved to be efficient, as all the groups showed statistically significant differences, except symmetric volunteers and presurgical symmetric subjects, as expected. This result demonstrates that automatic MSP construction could be used to classify subjects based on their asymmetry level and has clinical relevance. However, some outlier cases were found, as reported in Fig. 3 and in our qualitative analysis of 2 error cases. These outliers were due to automatic localization errors, which could be related to atypical morphology, CT scan acquisition properties or metal artifacts [8]. It should be noted that the CT scans of the volunteer subjects had a mean slice thickness twice that of the presurgical subjects, which may have affected landmark localization accuracy in this dataset. For clinical planning, an expert review of the annotations and MSP construction will always be needed.

In the implementation presented in this study, MSP construction was fully automated and took approximately 1 min per CT scan. Since the MSP construction was based on classical anatomical landmarks, we believe that a clinician could easily verify their localization and correct them if necessary, but we did not test this scenario. Thus, compared to other MSP construction methods based on morphometry or symmetry plane, the automatic landmark-based method has the advantage of being both fast and easy to understand anatomically for a clinician. However, our implementation remains limited to research purposes for now, as it has not yet been implemented in any clinical software. Like the other two methods, it still needs to be implemented in user-friendly software to facilitate clinical use and manual correction of annotations when needed. The main disadvantage of landmark-based MSP construction is its inability to adapt to skull base asymmetries, which can be particularly prevalent in patients with craniofacial syndromes [20]. For these patients, other methods, such as voxel-based morphometry [20] or symmetry plane [21–23] might be more reliable.

This study has some limitations. First, it was performed on CT scans rather than CBCT scans, which are more commonly used for 3D cephalometric studies. This limitation was due to the fact that our deep learning model was trained only on CT scans, which is the imaging modality used for orthognathic surgery planning in our department. Similarly, the dataset did not include subjects with temporary teeth because it consisted only of adult scans. Recent research reports show that state of the art deep learning models trained on CBCT scans for landmark localization can perform with comparable accuracy to the model used in this study [24, 25]. As a result, automatic MSP construction should show similar reliability on CBCT scans when using a dedicated deep learning model. Second, the dataset was divided into 4 categories based on the qualitative assessment of one experienced operator. An evaluation by multiple clinicians or based on more clinical data may have changed these categories. Third, our repeatability and reproducibility study was conducted on only 19 subjects and may not capture the full range of dentofacial deformities found in the dataset.

In this work, we focused on evaluating MSP construction for measuring mandibular asymmetry, as this is a recurring clinical problem and 3D data provide clinically significant information when compared with 2D radiographs. This is a first step towards a complete 3D cephalometric analysis, which remains a major clinical challenge and requires more clinical data [24].

## Conclusion

The proposed MSP construction was shown to be fast and fully automatized in our research implementation. It was shown to be as reliable as manual construction, and clinically relevant in assessing the mandibular asymmetry of 368 head CT scans. Once implemented in a clinical software, this method could provide clinicians a fast and easy solution for CT scans orientation and mandibular asymmetry evaluation.

## Data Availability

The data that support the findings of this study are available from the corresponding author upon reasonable request.No datasets were generated or analysed during the current study.
